# Dietary Fiber from Navel Orange Peel Prepared by Enzymatic and Ultrasound-Assisted Deep Eutectic Solvents: Physicochemical and Prebiotic Properties

**DOI:** 10.3390/foods12102007

**Published:** 2023-05-16

**Authors:** Liling Zhou, Jiaqian Luo, Qiutao Xie, Lvhong Huang, Dan Shen, Gaoyang Li

**Affiliations:** 1 Longping Branch, College of Biology, Hunan University, Changsha 410125, China; 2 Hunan Agricultural Product Processing Institute, Hunan Academy of Agricultural Sciences, Hunan Provincial Key Laboratory for Fruits and Vegetables Storage Processing and Quality Safety, Changsha 410125, China

**Keywords:** orange pomace, dietary fiber, deep eutectic solvent, physicochemical, prebiotic activity

## Abstract

Dietary fiber (DF) was extracted from navel orange peel residue by enzyme (E-DF) and ultrasound-assisted deep eutectic solvent (US-DES-DF), and its physicochemical and prebiotic properties were characterized. Based on Fourier-transform infrared spectroscopy, all DF samples exhibited typical polysaccharide absorption spectra, indicating that DES could separate lignin while leaving the chemical structure of DF unchanged, yielding significantly higher extraction yields (76.69 ± 1.68%) compared to enzymatic methods (67.27 ± 0.13%). Moreover, ultrasound-assisted DES extraction improved the properties of navel orange DFs by significantly increasing the contents of soluble dietary fiber and total dietary fiber (3.29 ± 1.33% and 10.13 ± 0.78%, respectively), as well as a notable improvement in the values of water-holding capacity, oil-holding capacity, and water swelling capacity. US-DES-DF outperformed commercial citrus fiber in stimulating the proliferation of probiotic *Bifidobacteria* strains in vitro. Overall, ultrasound-assisted DES extraction exhibited potential as an industrial extraction method, and US-DES-DF could serve as a valuable functional food ingredient. These results provide a new perspective on the prebiotic properties of dietary fibers and the preparation process of prebiotics.

## 1. Introduction

Dietary fiber (DF), made up of indigestible polysaccharides, can provide various benefits, such as regulating intestinal microfloral composition, lipid metabolism, and fecal volume [1]. DF could be classified into two primary categories based on its solubility: insoluble dietary fiber (IDF) and soluble dietary fiber (SDF). IDF primarily comprises cellulose, hemicellulose, and lignin, while SDF mainly comprises pectin, some hemicellulose, and non-cellulosic polysaccharides, such as inulin. Fermentation of DF by intestinal microflora could produce short-chain fatty acids that modulate the intestinal pH and suppress pathogenic bacterial growth. The inclusion of DF in the diet has been shown to stimulate *Bifidobacteria* growth, which is closely associated with intestinal health and is considered beneficial for human health [2,3]. Previous research has demonstrated that SDF possesses more important physiological functions and superior physicochemical properties than IDF, allowing for a wider range of applications in food. This is attributed to its hydrophilic, non-crystalline nature, which enables easy wetting by gastrointestinal water and facilitates the formation of viscous colloidal dispersions or gels upon hydration. Additionally, SDF is more readily fermentable by the intestinal microbiota [4,5].

Dietary fibers derived from fruits are typically high in human health-beneficial biological activities [6]. Citrus is the most produced fruit globally. Its potential functional and nutritional benefits have led citrus to be listed as one of the most promising functional foods [7]. Citrus dietary fiber is characterized by a unique microstructure and a high content of SDF [8]. As the primary type of citrus DF, orange dietary fiber has exhibited a broad range of applications in the food industry. Adding pectin-rich orange fibers to yogurt as fillers can stabilize the casein network and extend shelf life, owing to pectin’s sensitivity to calcium [9]. Additionally, the high oil-holding capacity of orange dietary fiber makes it an appropriate fat substitute in food, suppressing obesity [10]. Orange peel residue is the primary waste product in the orange processing industry, accounting for approximately 55–60% of fresh fruit weight [11], and is an abundant source of dietary fiber. If this waste is discarded rather than processed into added-value products, it harms the environment and causes economic losses. Therefore, developing innovative methods for processing and extracting orange DF from orange peel residue can potentially improve the utilization efficiency of orange resources and promote efficient orange processing.

In dietary fiber extraction methods, enzymatic extraction offers several advantages over chemical extraction (acid and alkali), such as environmental protection and low energy consumption. However, the loss of polysaccharides during the hydrolysis and pretreatment processes would reduce the DF yield [12,13]. Deep eutectic solvent (DES) is a low melting point mixture formed by hydrogen bonding between a hydrogen bond donor (HBD) and acceptor (HBA), which offers advantages over conventional organic solvents, including low toxicity, noninflammability, low-cost synthesis, and the fact that it does not require purification, making it suitable for large-scale production. DES, or nature deep eutectic solvent (NADES) has been used for the extraction of bioactive compounds such as alkaloids [14], anthocyanins [15], phenyletanes and phenylpropanoids [16], saponins [17], polysaccharides [18], carotenoids, and hydrophilic ascorbic acid [19]. Choline chloride (ChCl) is the most common HBA in the preparation of DESs. ChCl-based DESs have been widely used for extracting pectin, hemicelluloses, and lignin [20,21,22,23,24]. The cellulose content in orange dietary fiber is substantially greater than other components, and lignin significantly impedes cellulose extraction from natural resources [25]. As a result, promoting lignin dissolution can significantly enhance the extraction rate of orange dietary fiber. Previous research indicated that ultrasound-assisted extraction could significantly improve the extraction effect of DES. Ultrasound-assisted DES extraction has the potential to enhance the sustainability of natural product extraction by reducing inputs of energy and labor and disrupting plant cell wall structure by ultrasonic cavitation, leading to an increased extraction yield [26,27]. Polysaccharides extracted using choline chloride and lactic acid-based deep eutectic solvents (DES) at a molar ratio of 1:2 exhibit significantly higher antioxidant and biological activity levels than polysaccharides obtained via hot water extraction [28]. The utilization of DES to treat orange pomace, remove lignin, and combine it with other treatment procedures to facilitate the conversion of cellulose to soluble dietary fiber can enhance the physiological functionality of orange dietary fiber.

The present study employed ultrasound-assisted DES extraction to extract DF from navel orange pomace and compare it with enzymatically extracted DF. The physicochemical properties of DFs were characterized, and the potential prebiotic activity was evaluated using three *Bifidobacterium* strains. The study was aimed at offering valuable insights into the utilization of orange processing by-products and the development of new prebiotics.

## 2. Materials and Methods

### 2.1. Materials and Chemicals

Fresh navel oranges (*Citrus sinensis* (L.) Osbeck) were obtained from the local marketplace in Xinning County, Hunan Province, China, ensuring they were free from damage or decay. The peel was washed and subsequently dried using a hot air vacuum dryer for 48 h at 60 °C, resulting in a moisture content of 6.48%. Afterward, the dried sample underwent grinding and was sieved using an 80-mesh strainer to obtain the orange powder. The powder was defatted in 90% ethanol for 30 min, washed with deionized water, dried, and stored in a desiccator until needed.

Choline chloride (purity > 98%) and lactic acid (purity ~92%, moisture content ~8%) were from Shanghai Hushi Laboratory Equipment Co., LTD., Shanghai, China. Heat-stable α-amylase (EC 3.2.1.1, 4000 µ/g), amyloglucosidase (EC 3.2.1.3, 100 µ/mg), and papain (EC 3.4.22.2, 800 µ/mg) were purchased from Shanghai Ryon Biological Technology Co., LTD., Shanghai, China. KBr (Sinopharm Chemical Reagent Co., Ltd., Shanghai, China) was spectral grade, and all other chemicals were analytical grade.

### 2.2. Preparation of DES

According to the literature, the selected DES consisted of a molar ratio of 1:2 of choline chloride to lactic acid [29,30]. The mixture was stirred until a uniform and transparent liquid was formed at 60 °C and brought to room temperature. The viscosity of DES was reduced by adding deionized water in a ratio of 20% *v*/*v*.

### 2.3. Preparation of Orange Dietary Fiber (DF)

#### 2.3.1. Enzymatic Extraction

Based on the literature [8], orange peel powder (10 g) was mixed with 100 mL deionized water and sequentially treated with heat-stable α-amylase (0.5 g, pH 5.5, 90–95 °C, 1 h), amyloglucosidase (0.3 g, pH 4.2, 60 °C, 1 h), and papain for hydrolysis (0.36 g, pH 6–7, 55 °C, 2 h). To inactivate the enzymes, the reaction mixture was heated in a boiling water bath for 15 min, then cooled to room temperature and precipitated with 95% ethanol in a four-fold volume at 4 °C overnight. The precipitated mixture was centrifuged at 5500× *g* for 15 min, and the resulting precipitate was freeze-dried to obtain E-DF.

#### 2.3.2. DES Extraction

An amount of 10 g orange peel powder and 200 mL of DES (1:20, *w*:*v*) were stirred at 120 °C for 3 h, and the beaker of the mixture was immersed in cold water until cooled to room temperature (25 °C ± 2 °C), then centrifuged at 5500× *g* for 15 min. The DF was precipitated with 95% aqueous ethanol (four volumes, 4 °C overnight), centrifuged again, washed with 70% aqueous ethanol and acetone until the washes were colorless [29], and then freeze-dried to obtain DES-DF.

#### 2.3.3. Ultrasound-Assisted DES Extraction

The extraction was performed as in Section 2.3.2, except that the orange peel powder and DES were mixed, then ultrasonicated for 15 min (power 300 W, pulsed action, 2 s on, 2 s off) to obtain US-DES-DF.

### 2.4. Physicochemical Properties

#### 2.4.1. Dietary Fiber Composition

Total dietary fiber (TDF) and soluble dietary fiber (SDF) were determined according to the AOAC method 991.43. Pectin content was measured by the colorimetric method [31]. Cellulose, hemicellulose, and lignin were measured by the method described previously [32].

#### 2.4.2. Proximate Composition

The moisture content was determined by drying the samples to a constant weight in an oven (DHG-9053A, Shanghai Jinghong Co., Ltd., Shanghai, China) set at 105 °C. The protein content was determined by the Kjeldahl method. Ash content was determined using a muffle furnace (SX-2-8-10, Changsha Yuandong Electric Furnace Co., Ltd., Shanghai, China) at 550 °C. The total polyphenols were determined by the Folin–Ciocalteu reagent assay using gallic acid as a standard [33]. The total carbohydrate content was determined by the phenolsulfuric acid method [34].

#### 2.4.3. Hydration and Adsorption Capacities

Water holding capacity (WHC), water swelling capacity (WSC), and rapeseed oil holding capacity (OHC) were determined as described previously [35].

### 2.5. Structural Analysis

#### 2.5.1. Scanning Electron Microscopy (SEM)

The microstructure of DF samples was observed through scanning electron microscopy (SEM; EVO LS10, Carl Zeiss, Dresden, Germany) after fixation and gold sputtering following the drying process.

#### 2.5.2. Fourier-Transform Infrared Spectroscopy (FT-IR)

Dried orange fiber samples were homogenized with KBr powder and ground evenly with an agate mortar. Disks were formed by pressing the mixture, which was then scanned over a wavenumber range of 400–4000 cm^−1^ using a Nicolet iS5 spectrometer (Thermo Fisher Scientific, Waltham, MA, USA) to obtain the FT-IR spectra.

#### 2.5.3. X-ray Diffraction (XRD)

XRD patterns were recorded on an X-ray diffractometer (D8 Advance, Bruker, Billerica, MA, USA). The operating conditions were 40 kV and 40 mA with Cu radiation. The samples were scanned at 2θ values spanning 5–50°. Crystallinity was calculated as described previously [8].

#### 2.5.4. Thermogravimetric Analysis (TGA)

Thermogravimetric analysis was determined using a Netzsch STA 449 F3 Jupiter thermal analyzer (Selb, Germany) under a nitrogen atmosphere. The samples were heated from 25 °C to 900 °C at a 10 °C/min rate during the analysis.

### 2.6. Prebiotic Activity Determination

The prebiotic activities of E-DF and US-DES-DF were determined in vitro by measuring the relative growth rates of selected *Bifidobacteria* strains as described previously [36], with minor modifications. The prebiotic activities of DFs were compared to those of commercial orange fiber (MG-DF, purchased from Xi’an Muguo Biotechnology Co., LTD., Xi’an, China) and the soluble prebiotic fructooligosaccharides (FOS) (Shanghai Hushi Laboratory Equipment Co., LTD., Shanghai, China), utilizing a pure culture medium as a negative control (CK).

#### 2.6.1. Bacterial Strains and Growth Conditions

*Escherichia coli* K12 was selected as a representative enteric species for comparison, and *Bifidobacterium infantis* ATCC 15697, *B. bifidum* ATCC 29521, and *B. adolescentis* ATCC 15703 (all from Guangdong Microbial Strain Preservation Center, Guangzhou, China) were chosen as representative probiotic strains. Each bacterial powder was activated by incubation for 48 h at 37 °C, with the *Bifidobacteria* being cultured anaerobically using an anaerobic pack from Mitsuba Aerobics Chemical Company, Japan. *E. coli* K12 was grown in a Luria–Bertani (LB) medium with tryptone (10 g), yeast extracts (5 g), and NaCl (5 g) per 1 L of distilled water. The components of the *Bifidobacteria* medium were as follows: soybean peptone (5 g), tryptone (5 g), yeast extract (10 g), salt solution (40 mL), L-cysteine (0.5 g), 0.1% resazurin (1 mL), and distilled water (1000 mL). The preparation of salt solutions: CaCl_2_ (0.2 g), MgSO_4_·7H_2_O (0.48 g), K_2_HPO_4_ (1 g), KH_2_PO_4_ (1 g), NaHCO_3_ (10 g), NaCl (2 g), and distilled water (1000 mL). The culture medium was sterilized for 15 min at 121 °C before use.

#### 2.6.2. Bacteria Growth Curve and pH Determination

Fructooligosaccharides or orange fiber (MG-DF, E-DF, US-DES-DF) (2%, *w*/*v*) was used as the only carbon source for the *bifidobacteria’s* growth, and the cultures were incubated anaerobically using an anaerobic airbag. Growth curves were determined by measuring OD_600_ at 4-hour intervals within 24 h of fermentation. Additionally, 5 mL of samples were collected at the start and endpoints of fermentation to assess pH.

#### 2.6.3. Prebiotic Index (PI)

Bacterial growth was evaluated by measuring colony-forming units (CFU/mL). PI is the ratio of the growth of bacteria on prebiotics to that on fructooligosaccharides. A PI > 1 indicates that the sample increases bacterial growth. PI was calculated as follows:

(1) $$\mathrm{PI}=\frac{CFU_{DF}}{CFU_{CK}}$$

where PI is the prebiotic index, *CFU_DF_* is the logarithm of growth in the prebiotic medium for 24 h (CFU/mL), and *CFU_CK_* is the logarithm of growth in the control medium for 24 h (CFU/mL).

#### 2.6.4. Prebiotic Activity Score (PA)

Substrates with high prebiotic activity fractions support good growth of probiotics, and colony-forming units (CFU/mL) are similar to those when growing on fructooligosaccharides. The PA was calculated as described previously [5]:

(2) $$\mathrm{PA}=\frac{{\left({LogP}_{24}-{LogP}_{0}\right)}_{DF}}{{\left({LogP}_{24}-{LogP}_{0}\right)}_{FOS}}-\frac{{\left({LogE}_{24}-{LogE}_{0}\right)}_{DF}}{{\left({LogE}_{24}-{LogE}_{0}\right)}_{FOS}}$$

where PA is the prebiotic activity score and *LogP* is the growth logarithm of probiotics cultured on prebiotics and fructooligosaccharides for 24 h (*P_24_*) and 0 h (*P_0_*). *LogE* is the growth logarithm of *Escherichia coli* K12 cultured on prebiotics and fructooligosaccharides for 24 h (*E_24_*) and 0 h (*E_0_*) (CFU/mL).

### 2.7. Statistical Analysis

Three independent measurements were taken, and the results were presented as the mean ± standard deviation. A single-factor analysis of variance (ANOVA) was performed on the data using SPSS 18.0 statistical software, incorporating Duncan’s multiple comparison method, at a significance level of *p* < 0.05.

## 3. Results and Discussion

### 3.1. Physicochemical Properties

#### 3.1.1. Extraction Yield and Chemical Composition

Table 1 presents the extraction yield of dietary fiber samples obtained from navel orange pomace (E-DF, DES-DF, and US-DES-DF). The results indicated that the extraction yield of US-DES-DF was significantly higher than that of DES-DF and E-DF, which was likely due to the loose structure of the DF sample under the effect of ultrasound, leading to enhanced interaction between the DES and the sample powder. Ultrasound treatment played a crucial role in enhancing extraction efficiency by modifying the structure of samples by creating cavitation bubbles and shear forces that increase the solvent penetration into the sample, which led to improved dissolution rates and yield enhancement. Furthermore, the cavitation phenomenon generated microstreams and microjets in the solvent, which helped break down the molecular bonds and reduce the particle sizes of the sample, resulting in improved solubilization and yield enhancement. As a result, it could be inferred that ultrasound-assisted deep eutectic solvent extraction (US-DES) improved the DES’s accessibility to the sample matrix and promoted effective extraction of DFs, resulting in a higher yield of US-DES-DF when compared to DES-DF and E-DF. The results suggest that US-DES has significant potential for improving the efficiency and yield of dietary fiber extraction.

Orange fiber mainly consists of insoluble dietary fiber (IDF) [8], with low fermentability and limited probiotic utilization. In contrast, soluble dietary fiber (SDF) provides significant physiological benefits and generally exhibits superior physicochemical properties compared to IDF [37,38]. Table 1 presents the chemical composition of orange peel and orange dietary fiber, indicating that the total dietary fiber (TDF), SDF, and IDF contents in the orange peel were significantly lower than those in the three navel orange fibers. This finding suggests that the purity of dietary fiber increases after removing alcohol-soluble materials from navel orange peel through different treatments [35]. Furthermore, the TDF and IDF contents were significantly higher in DES-DF than in E-DF, indicating that DES extraction effectively extracts IDF from navel orange peel and increases the TDF content in the final products. However, the effect of DES on SDF content was insignificant. The SDF contents of the three orange fibers exceeded those reported for lemon seed and cherry kernel [39]. US-DES-DF displayed the highest levels of total dietary fiber (TDF) and SDF at 87.00 ± 0.59% and 22.11 ± 1.43%, respectively. The findings suggest that ultrasound treatment may synergize with DES to break IDF into SDF. This effect was potentially due to the ability of ultrasound treatment to destroy the cellulose structure in orange peel, promoting the transformation of insoluble dietary fiber to soluble dietary fiber [3]. The results demonstrate the impact of various treatments on the quality and composition of the dietary fiber in navel oranges.

The IDF/SDF ratio is crucial in determining the physiological functions of dietary fiber. In the food industry, dietary fiber with a proposed IDF/SDF ratio of 2:1 is preferred for its superior quality. The results indicated that US-DES-DF approached this ratio, and enzymatic treatment improved the IDF/SDF ratio in orange peel. Conversely, DES treatment yielded more IDF, leading to a higher IDF/SDF ratio than in orange peel, which could be resolved through a combined ultrasound treatment.

As shown in Table 1, the pectin content in the three orange fibers was significantly higher than that of OP, possibly owing to the enzymatic or chemical mechanical treatment that activated the original pectin, resulting in the conversion of pectin to its water-soluble form. Furthermore, the gradual decrease in system pH caused by the liberation of acid from the orange peel could account for the lack of a significant impact of treatment on pectin degradation [35]. Cellulose and hemicellulose contents in OP were significantly lower than those in navel orange fibers due to enzymatic, chemical, and mechanical treatments that led to the degradation of non-fiber contents and increased the proportion of cellulose and hemicellulose. There was no significant difference in lignin content between OP and E-DF. However, a profound reduction in lignin was detected in DES-DF and US-DES-DF, indicating that DES can dissolve lignin effectively

Dietary fiber possesses potent antioxidant activity by associating with polyphenols via ionic, covalent, or hydrogen bonds [40]. The total polyphenols in the samples were determined using the Folin–Ciocalteau method, with results presented as gallic acid equivalent (GAE). The total polyphenol content in Navel orange peel was 28.63 ± 1.62 mg GAE/g, which was slightly higher than that of Thomson Navel (25.60 ± 0.23 mg GAE/g DM) [41] reported in previous research but significantly lower than that reported by Gorinstein Shela et al. (179 ± 10.5 mg/g) [42]. Specific varieties, planting environments, and harvest seasons, among other factors, might account for this difference in the total polyphenol content. Some polyphenols, such as anthocyanins and flavonoids, are less stable under high-temperature conditions and may undergo degradation, resulting in reduced polyphenol content. On the contrary, catechins and some other polyphenols show greater stability at high temperatures. Ultrasound-assisted DES has been shown to lead to structural changes in the plant matrix, allowing for enhanced solvent penetration and yielding a higher concentration of polyphenols [43]. This might have contributed to the higher total polyphenol content observed in US-DES-DF.

The proximate composition analysis showed no significant differences in moisture, protein, or total carbohydrate content among the three orange dietary fibers. However, the moisture content in orange peel was significantly higher than in the orange dietary fibers, likely due to variances in the drying techniques. The protein content of DFs was similar to the results reported by Zhang et al. [44]. Additionally, the ash content in the navel orange fibers was higher than in the orange peel, which may be caused by material degradation from enzyme treatment and ions introduced by chemical reagents. The results were similar to those reported by Wang et al. [45].

#### 3.1.2. Water-Holding Capacity (WHC), Water Swelling Capacity (WSC), and Oil-Holding Capacity (OHC) of Orange DFs

**Figure 1:**
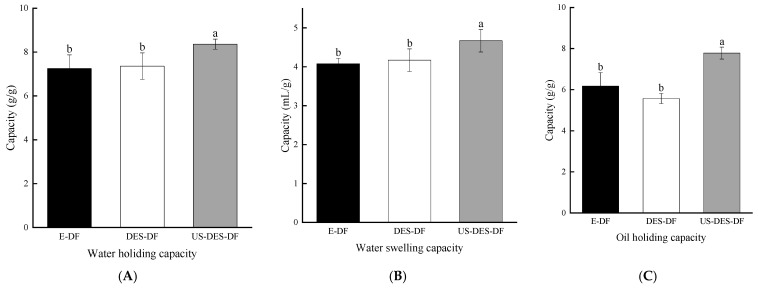
Water-holding capacity (WHC), water swelling capacity (WSC), and oil-holding capacity (OHC) of E-DF (**A**); DES-DF (**B**); and US-DES-DF (**C**). Different lowercase letters indicate significant differences (*p* < 0.05), while the same letter indicates no significant difference (*p* > 0.05) (Duncan’s test).

Water-holding capacity (WHC) is a crucial parameter indicating the ability of dietary fibers to retain moisture. High WHC values can confer a thicker texture and greater stability to food products. The number and nature of water-binding sites and the fiber structure mainly determine the WHC of DFs. As Shown in Figure 1, there is no significant difference in WHC values between E-DF and DES-DF (7.25 ± 0.62 g/g and 7.36 ± 0.60 g/g, respectively). DES treatment increased the WHC from (E-DF) to (DES-DF; Figure 1), and ultrasound-assist DES further increased WHC (8.52 ± 0.23 g/g), which was probably linked to the SDF increase (Table 1). Furthermore, ultrasound cavitation generates greater hydrodynamic shear forces, which expose additional hydrophilic sites and chemical groups, leading to increased fiber porosity and enhanced DF hydrophilicity [46]. Consequently, the facile access of water molecules to the fiber network and a higher amount of water retention occurs, resulting in increased WHC values.

The capillary structure of dietary fibers facilitates water retention, which affects water swelling capacity (WSC), representing the fiber’s ability to absorb water, expand, and impact its functionality in food applications. The WSC of DFs depends on their structure and properties, which can influence the extent of water absorbance and expansion. As shown in Figure 1, there was no significant difference in WSC between E-DF and DES-DF. US-DES-DF exhibited the highest WSC (4.67 ± 0.29 mL/g). This can be attributed to the ultrasound-induced disruption of the fiber structure during the extraction process. The acoustic waves generated by ultrasound can disrupt the intermolecular networks of DFs, promote water molecule interaction with the polysaccharides in the fiber network, and facilitate swelling in the presence of water.

Oil-holding capacity (OHC) indicates how much oil or fat can be physically bound and held by dietary fiber, reducing absorption during food digestion. Dietary fiber with high OHC values could serve as a fat substitute in the food industry, offering a healthier alternative to conventional high-fat food products. The OHC of DFs depends on the type, properties, and extraction method of the fiber. As illustrated in Figure 1, US-DES-DF had a higher OHC (7.78 ± 0.29 g/g) than E-DF (6.18 ± 0.65 g/g) and DES-DF (5.57 ± 0.25 g/g). Previous studies have demonstrated that enzymatic extraction of soluble dietary fibers (SDF) could generate higher OHC values than alkaline or acid extraction approaches [12]. This evidence suggests that the US-DES extraction method may be a promising approach for increasing the OHC of dietary fibers.

### 3.2. Structural Analysis

#### 3.2.1. SEM

**Figure 2:**
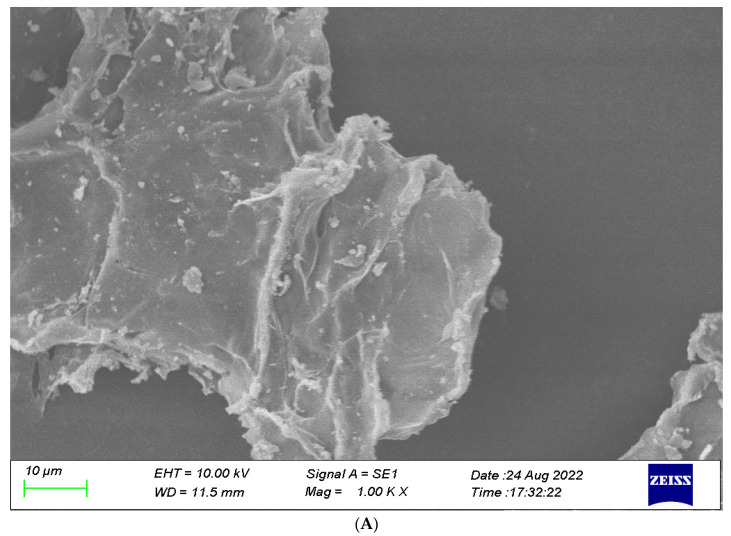

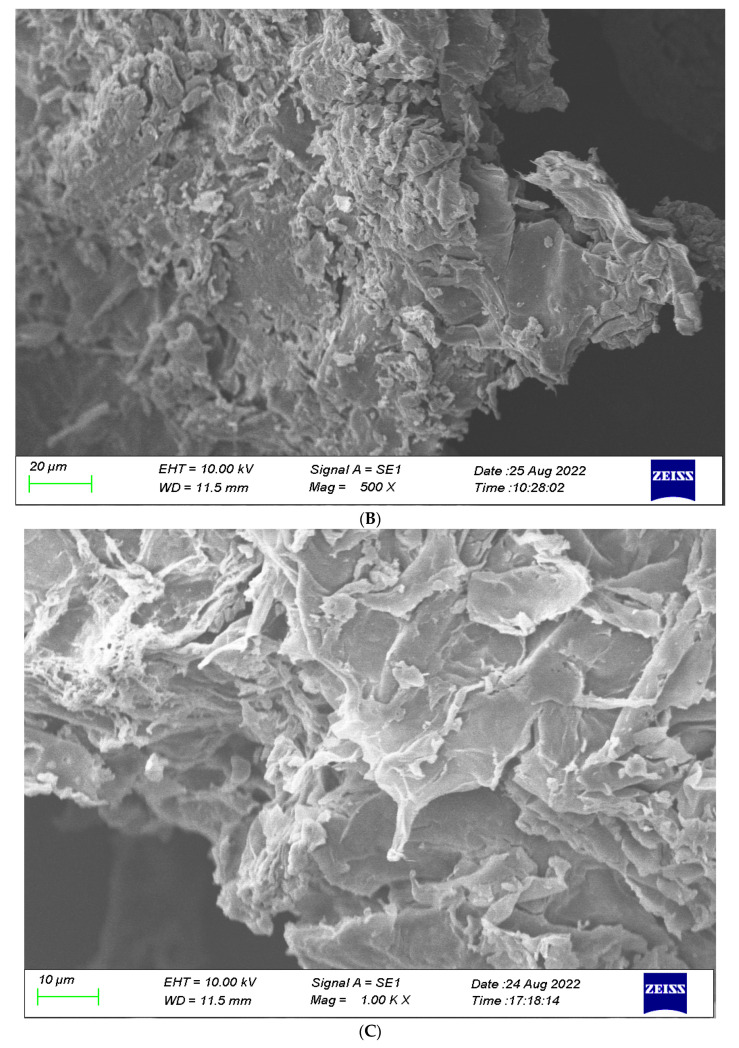
SEM images of E-DF (**A**), DES-DF (**B**), and US-DES-DF (**C**). Magnification: 500× and 1000×.

The dietary fiber (DF) microstructure determines its physiological function. A looser and more porous microstructure of DF would enhance WHC, WSC, and OHC. SEM analysis of E-DF illustrated its surface as compact and relatively smooth, with closely arranged fibrous matter and small fragments adhering to particle surfaces (Figure 2A). The surface of DES-DF was much rougher and irregular (Figure 2B). This structure increased the specific surface area of the particles, leading to an enhanced absorption capacity for water and oil. Similarly, the surface of US-DES-DF exhibited noticeable irregularities and multilayer structures with a high density of irregular filaments (Figure 2C), leading to an increased specific surface area that exposes more water-binding sites [47]. Cellulose and hemicellulose degradation resulted in cracks on the particle surface of US-DES-DF. Moreover, ultrasound treatment could degrade cellulose and hemicellulose [48], altering the structure, hydration, and absorption capacity of US-DES-DF. This observation is consistent with earlier research on orange-derived DFs, where ultrasonic treatment induced comparable structural modifications and elevated functional properties. Similarly, changes in structure were also witnessed in lemon fiber after ultrasound treatment [49].

#### 3.2.2. FT-IR

**Figure 3:**
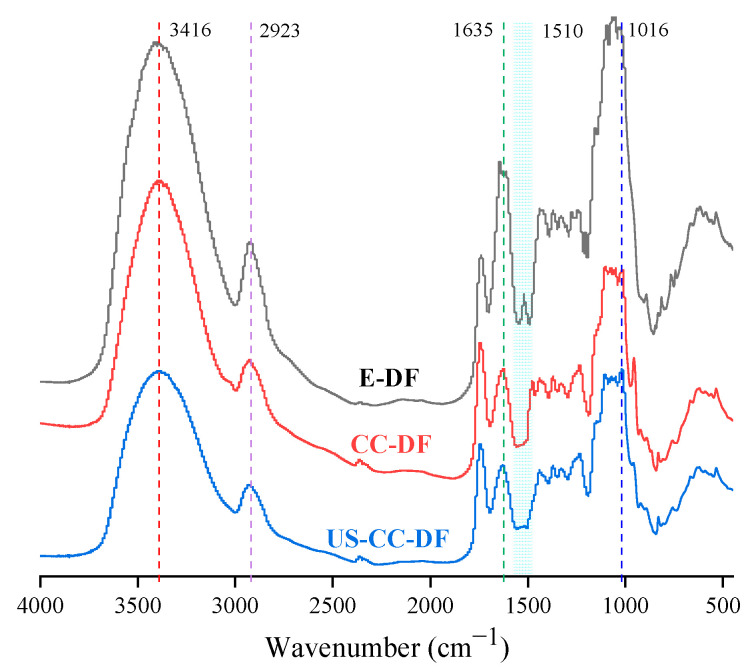
FT-IR spectra of E-DF, DES-DF, and US-DES-DF.

The FT-IR spectra of the DFs were determined (Figure 3). The spectra and individual peak shapes of the three orange fibers were similar, indicating comparable chemical compositions. The large O–H stretching vibration absorption peak (3200–3500 cm^−1^) indicated the presence of abundant hydrogen bonds [13]. The polysaccharide’s characteristic methyl and methylene C–H bond stretching vibrations were observed at 2923 cm^−1^, indicating a typical polysaccharide structure [50]. The C–O–C stretching vibration at 1016 cm^−1^ was a typical sugar aldehyde group [51]. The lignin aromatic phenyl ring absorption at 1510 cm^−1^ was only present in the E-DF spectrum, indicating that DES treatment dissolved the lignin. Although the E-DF, DES-DF, and US-DES-DF spectral peak intensities differed significantly, the overall peak type and position were remarkably similar, implying that the DF treated with DES or ultrasound-assist DES did not generate any new functional groups. The differences in the intensity of absorption peaks indicate that the physicochemical properties and functional activities of DFs could be diverse.

#### 3.2.3. XRD

**Figure 4:**
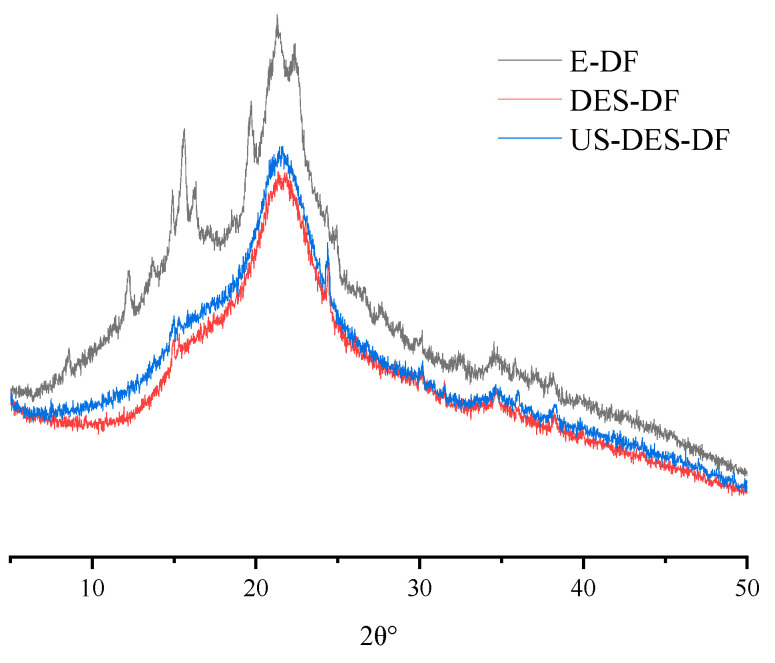
XRD spectra of E-DF, DES-DF, and US-DES-DF.

The properties of DF were greatly influenced by crystallinity, and a decrease in crystallinity may lead to increased extensibility and softness of the orange fiber. In contrast, increasing crystallinity may enhance thermal stability [35]. The XRD spectra of the three DFs were similar overall, signifying a comparable crystal structure (Figure 4). The E-DF spectra contain minor peaks that could result from enzymatic hydrolysis, causing cellulose breakdown in specific dietary fiber regions [52]. The central peak in all three spectra was around 22°, indicating the presence of a cellulose I crystal configuration. E-DF possessed the highest crystallinity index (36.70%), followed by DES (31.48%). Nevertheless, US-DES-DF exhibited a significantly reduced crystallinity index of 29.73% following ultrasound treatment, which may have resulted from cellulose chain disruption caused by ultrasonic cavitation.

#### 3.2.4. TGA

**Figure 5:**
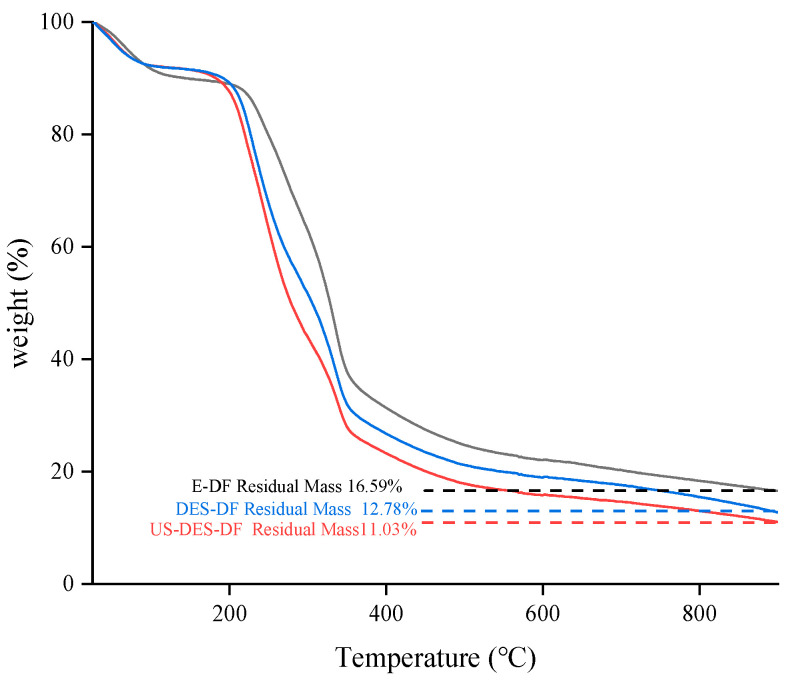
Thermogravimetric melting/decomposition curves of E-DF, DES-DF, and US-DES-DF.

Thermal stability is crucial when applying DF, as heating is integral to most food processes. The thermal decomposition of orange fiber occurs in three stages (Figure 5). At the initial stage (60−160 °C), the evaporation of intramolecular free and crystal-bound water resulted in water loss. During the second stage of the process (160−250 °C), hemicellulose and pectin undergo thermal depolymerization, while the crystalline regions of cellulose undergo degradation. E-DF, DES-DF, and US-DES-DF had mass losses of 10.02%, 23.68%, and 28.09%, respectively. The structure of US-DES-DF was loosened by ultrasound-assisted DES extraction. Consequently, soluble dietary fiber (SDF) content in US-DES-DF was higher than in E-DF and DES-DF (Table 1), mainly consisting of pectin and some hemicellulose, resulting in the highest weight loss in US-DES-DF. The decomposition of some lignin and complex polymers was primarily responsible for the mass loss at the third stage (> 250 °C). After three stages, the residual masses of E-DF, DES-DF, and US-DES-DF were 16.59%, 12.78, and 11.03%, respectively. The amount of residue may be influenced by the different extraction methods and crystallinities [35]. The structural characteristics of DES and US-DES-DF reveal a looser arrangement as compared to E-DF, wherein the crystallinity of E-DF manifests as the highest among the three types of dietary fibers. This crystal structure of E-DF could be attributed to its superior thermal stability when subjected to high temperatures, while DES-DF and US-DES-DF exhibit a more extensive thermogravimetric decomposition.

### 3.3. Prebiotic Activity

Based on the structural and physicochemical characterization results, ultrasound-assisted DES extraction of orange fiber was expected to be more suitable for probiotic fermentation than DES extraction. The US-DES-DF had significantly higher WHC, WSC, OHC, and SDF content than the DES-DF. A positive correlation exists between prebiotic activity and WHC, OHC, and WSC. Additionally, SDF exhibits greater ease of colonic fermentation than IDF and can serve as a valuable probiotic resource [3,53,54]. Consequently, US-DES-DF was selected for an in vitro *Bifidobacteria* proliferation experiment.

#### 3.3.1. Growth Curves of Bifidobacteria Strains

**Figure 6:**
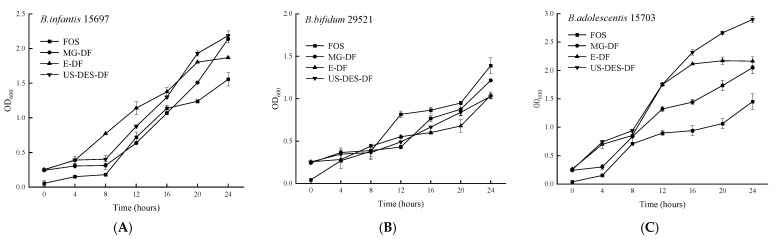
Growth curves (OD_600_) of *B. infantis* ATCC 15697 (**A**); *B. bifidum* ATCC 29521 (**B**); and *B. adolescentis* ATCC 15703 (**C**), in the presence of fructooligosaccharides (FOS), commercial orange fiber (MG-DF), enzyme-extracted orange fiber (E-DF), or ultrasound-assisted DES-extracted orange fiber (US-DES-DF), as sole carbon sources.

An inadequate carbon source and increased medium acidity would cause a decrease in *Bifidobacteria* growth on DF as the sole carbon source after 24 h [55]. Therefore, this study monitored growth curves for up to 24 h. The 24-hour growth curves (OD_600_) of *Bifidobacteria* are shown in Figure 6. All three *Bifidobacterium* strains exhibited growth and proliferation on all four DFs as the sole carbon source, indicating that DFs could support *Bifidobacteria* growth in the human colon. Previous research also indicated that DFs could undergo fermentation by the colon microflora, stimulating the growth and activation of beneficial bacterial populations [56]. *B. bifidum* 29521 proliferated more rapidly in a FOS-containing medium than with the three orange DFs, which contain more insoluble fractions (~75%, Table 1), which must be broken down into mono- or oligo-saccharides before absorption. *B. adolescentis* 15703 displayed significantly greater proliferation rates within US-DES-DF than within E-DF, FOS, or MG-DF, indicating that ultrasound-assisted extraction likely contributed to the easier degradation of US-DES-DF.

Compared to E-DF and commercial dietary fiber (MG-DF), US-DES-extracted DF appeared to have a higher content of SDF and a larger surface area, providing superior probiotic effects. However, orange fiber was mainly made up of IDF, with ~25% of SDF. Unlike readily absorbed FOS, DF was required to be degraded into an absorbable form to stimulate the growth of beneficial bacteria [53]. However, DF was indigestible by human enzymes and broken down by colon microbial enzymes [57,58]. Thus, US-DES-DF remains a viable substrate for *Bifidobacterium* growth.

#### 3.3.2. The pH Changes after Fermentation of Bifidobacteria Strains in the Presence of DFs

**Figure 7:**
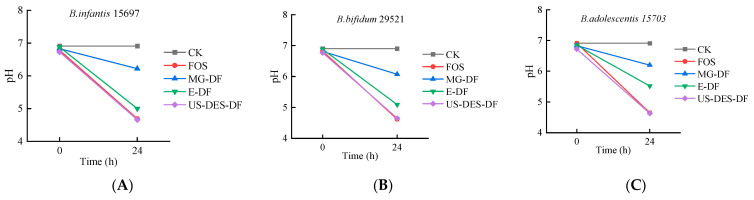
The pH changes after fermentation of *B. infantis* ATCC 15697 (**A**); *B. bifidum* ATCC 29521 (**B**); and *B. adolescentis* ATCC 15703 (**C**), in the presence of fructooligosaccharides (FOS), commercial citrus fiber (MG-DF), enzyme-extracted orange fiber (E-DF), or ultrasound-assisted DES-extracted orange fiber (US-DES-DF), as sole carbon sources, and no-carbon source control (CK).

Alterations in the pH of the intestinal environment could provide an indirect indication of changes in the composition of the intestinal microbiota and the fermentation conditions [59]. Figure 7 demonstrates a decrease in pH values across all three microbial strains undergoing fermentation with DFs, indicating the fermentative capacity of orange fiber or fructooligosaccharides (FOS) to serve as viable fermentation substrates for *Bifidobacteria* and facilitate the production of acids. FOS and US-DES-DF demonstrated the lowest pH levels (~4.6–4.7) among all three stains, followed by E-DF and MG-DF. The pH of the no-carbon source control (CK) remained largely unchanged, indicating minimal bacterial growth. The results suggested the prebiotic potential of orange fiber and FOS in promoting the growth of beneficial gut microbes and contributing to maintaining a healthy gut environment.

#### 3.3.3. Prebiotic Index of MG-DF, E-DF, and US-DES-DF

**Figure 8:**
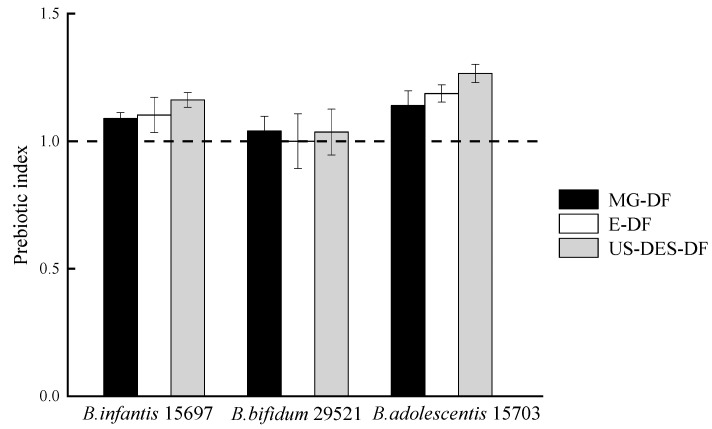
Prebiotic indexes of *B. infantis* ATCC 15697, *B. bifidum* ATCC 29521, and *B. adolescentis* ATCC 15703 grown on commercial citrus fiber (MG-DF), enzyme-extracted orange fiber (E-DF), or ultrasound-assisted DES-extracted orange fiber (US-DES-DF).

The prebiotic index (PI) value denotes the rate of probiotic strain growth in the presence of a prebiotic substance relative to FOS. A PI value < 1 indicated slower growth, and a PI value > 1 suggested faster growth. The three DFs exhibited no significant effect on the growth rate of *B. bifidum* 29521 (Figure 8). At the same time, DFs significantly stimulated the proliferation of *B. adolescentis* 15703 and *B. infantis* 15697, especially in the presence of US-DES-DF. The looser fiber structure and larger surface area of US-DES-DF appeared to facilitate the degradation of dietary fiber and enhance its ability to promote probiotic proliferation [60]. Although the prebiotic index of US-DES-DF (1.16–1.27) was much lower than that of a commercial prebiotic (7.22) [61], it could still be regarded as a prebiotic ingredient for functional food, owing to its high resistance to digestion and rapid clearance through the gastrointestinal tract [53]. It has been suggested that the beneficial effects of dietary fiber on gut health result from the balance between their resistance to digestion and their fermentability by the colonic microbiota. Therefore, DFs such as US-DES-DF, with their high resistance to digestion and significant ability to stimulate the proliferation of probiotics, may have the potential as a functional food ingredient to promote gastrointestinal health. Nevertheless, further research is needed to confirm the prebiotic potential of US-DES-DF and its health benefits.

#### 3.3.4. Prebiotic Activity Scores of MG-DF, E-DF, and US-DES-DF

**Figure 9:**
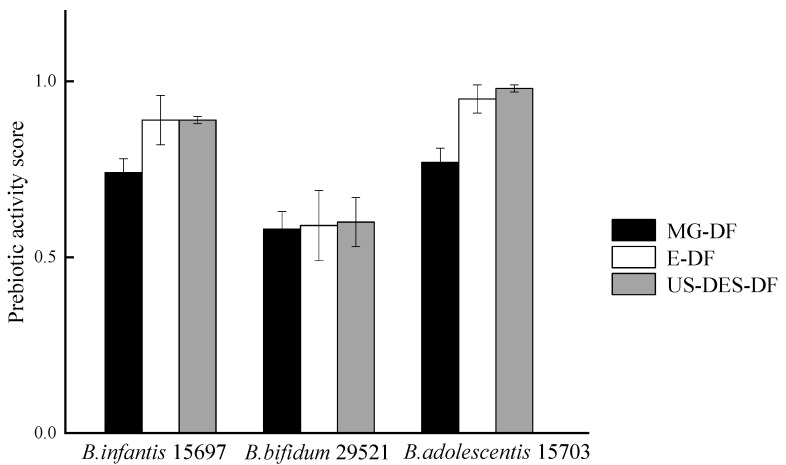
Prebiotic activity scores of *B. infantis* ATCC 15697, *B. bifidum* ATCC 29521, and *B. adolescentis* ATCC 15703 grown on commercial citrus fiber (MG-DF), enzyme-extracted orange fiber (E-DF), or ultrasound-assisted DES-extracted orange fiber (US-DES-DF).

Prebiotic supplementation could positively influence the composition and metabolic activities of the gut microbiota, leading to enhanced nutrient bioavailability, immune function, and disease prevention. The prebiotic activity scores (PA) of *B. infantis* 15697, *B. bifidum* 29521, and *B. adolescentis* 15703 were determined by assessing orange DF extract-induced growth stimulation compared to *E. coli* K12, where a positive PA value indicated that the DF sample could stimulate strain growth more effectively than *E. coli* K12. As shown in Figure 9, all PA values obtained were positive. *B. adolescentis* 15703 exhibited the highest PA value with US-DES-DF and E-DF (0.98 and 0.95, respectively), followed by *B. infantis* 15697 with US-DES-DF and E-DF (both 0.89). In contrast, the PAs of *B. bifidum* 29521 with all three DFs were similar (~0.59). Due to their resistance to hydrolysis by digestive enzymes within the small intestine, DFs could undergo fermentation by the colon microflora, resulting in the growth and activation of advantageous bacterial populations [62,63]. The findings indicated that US-DES-DF exhibits potential as a prebiotic that could effectively stimulate the growth and proliferation of probiotics.

## 4. Conclusions

In this study, orange dietary fiber (DF) was extracted by enzymatic (E-DF) and deep eutectic solvent (DES-DF), and its effects on structure, physicochemical, and in vitro prebiotic properties were evaluated. The results indicated that DES could improve lignin separation and increase DF yield. With ultrasound assistance, US-DES-DF had the highest proportion of SDF, water-holding capacity, oil-holding capacity, and water swelling capacity. Both E-DF and US-DES-DF were suitable sole carbon sources for *Bifidobacteria*. US-DES-DF had a more significant stimulatory effect on *Bifidobacteria* proliferation than E-DF and commercial orange fiber, demonstrating superior prebiotic activity. The results suggested that US-DES-DF has the potential to be used as a functional food to promote the proliferation of beneficial colon bacteria and maintain intestinal health. Ultrasound-assisted DES treatment was a safe and efficient extraction method that improved the functionality of orange fiber extracted from orange peel residue. The findings of this study present novel prospects for enhancing the utilization of orange resources and advancing prebiotic development.

## Figures and Tables

**Table 1:** Extraction yield and compositions of orange peel (OP), E-DF, DES-DF, and US-DES-DF.

	OP	E-DF	DES-DF	US-DES-DF
Extraction yield (%)	-	67.27 ± 0.13 ^c^	71.63 ± 1.56 ^b^	76.69 ± 1.68 ^a^
TDF (%)	65.40 ± 0.09 ^d^	76.88 ± 1.37 ^c^	82.96 ± 0.44 ^b^	87.00 ± 0.59 ^a^
SDF (%)	14.48 ± 0.61 ^c^	18.83 ± 0.10 ^b^	18.26 ± 0.89 ^b^	22.11 ± 1.43 ^a^
IDF (%)	50.92 ± 0.70 ^c^	58.05 ± 1.27 ^b^	64.70 ± 1.34 ^a^	64.89 ± 0.84 ^a^
IDF/SDF	3.52	3.08	3.54	2.93
Pectin (%)	12.60 ± 1.11 ^b^	13.46 ± 0.80 ^b^	13.58 ± 0.80 ^b^	15.75 ± 1.00 ^a^
Cellulose (%)	25.87 ± 0.89 ^d^	29.38 ± 1.36 ^c^	44.76 ± 0.69 ^a^	39.53 ± 0.61 ^b^
Hemicellulose (%)	14.21 ± 1.52 ^c^	16.42 ± 1.27 ^b^	17.33 ± 0.85 ^b^	22.71 ± 1.40 ^a^
Lignin (%)	8.77 ± 1.28 ^a^	9.48 ± 0.79 ^a^	0.92 ± 0.17 ^b^	0.99 ± 0.15 ^b^
Moisture (%)	6.48 ± 0.14 ^a^	2.88 ± 0.27 ^b^	2.86 ± 0.08 ^b^	2.91 ± 0.31 ^b^
Protein (%)	6.04 ± 0.44 ^a^	4.77 ± 0.55 ^b^	5.37 ± 0.36 ^b^	4.82 ± 0.87 ^b^
Ash (%)	3.83 ± 0.02 ^bc^	4.78 ± 0.27 ^a^	4.27 ± 0.27 ^b^	4.15 ± 0.65 ^b^
Total polyphenols (mg GAE/g)	28.63 ± 1.62 ^c^	29.63 ± 1.35 ^c^	35.78 ± 2.35 ^b^	41.22 ± 0.35 ^a^
Total carbohydrate	84.34 ± 0.34 ^a^	86.35 ± 1.98 ^a^	83.58 ± 1.05 ^a^	84.23 ± 2.66 ^a^

Different letters (a, b, c, and d) in the same row represent significant differences at *p* < 0.05 (Duncan’s test). TDF—total dietary fiber; SDF—soluble dietary fiber; IDF—insoluble dietary fiber.

## Data Availability

Data is contained within the article.
